# Epidemiological and Clinical Features of Dengue Infection in Adults in the 2017 Outbreak in Vietnam

**DOI:** 10.1155/2019/3085827

**Published:** 2019-11-07

**Authors:** Bui Vu Huy, Le Nguyen Minh Hoa, Dang Thi Thuy, Nguyen Van Kinh, Ta Thi Dieu Ngan, Le Van Duyet, Nguyen Thanh Hung, Ngo Ngoc Quang Minh, Nguyen Thanh Truong, Nguyen Van Vinh Chau

**Affiliations:** ^1^Hanoi Medical University, Hanoi, Vietnam; ^2^National Hospital for Tropical Diseases, Hanoi, Vietnam; ^3^Children's Hospital 1, Ho Chi Minh City, Vietnam; ^4^Hospital for Tropical Disease, Ho Chi Minh City, Vietnam

## Abstract

**Purpose:**

The clinical features and laboratory results of dengue-infected adult patients admitted to the hospital during the 2017 outbreak were analyzed in this study.

**Method:**

This is a cross-sectional study. 2922 patients aged 18 years or more with dengue fever in National Hospital for Tropical Diseases (NHTD) in the North and Hospital for Tropical Disease (HTD) in the South of Vietnam were recruited in this study.

**Result:**

Patients were admitted in the hospital around the year and concentrated from August to December, in 53/63 (84.0%) provinces in Vietnam, and patients in all ages were affected. The number of patients with dengue fever was 1675 (57.3%), dengue with warning signs 914 (31.3%), and severe dengue 333 (11.4%), respectively. Among patients with severe dengue, severe plasma leakage and dengue shock account for 238 (8.1%), severe organ impairment 73 (2.5%), and severe bleeding 22 (0.75%). The rate of mortality was 0.8%, and the outcome of dengue patients is worse in the elderly and people with underlying diseases.

**Conclusion:**

The 2017 dengue outbreak occurred in a larger scale than in the previous years in terms of time, location, and number of patients. More elderly patients were infected by dengue in this outbreak, and this may contribute to the mortality rate. Clinical manifestations of dengue patients in Southern Vietnam are more typical than the northern, but the rate of severe dengue is not different. The mortality risk and underlying conditions associated with dengue-infected elderly patients are worthy of further investigations in the future.

## 1. Background

According to the WHO, dengue is one of the mosquito-borne viral diseases that poses high medical burden in many regions worldwide recently. Before 1970, limited number of countries reported severe dengue epidemics [1]. However, the disease is now endemic in more than 100 countries in the regions of Africa, America, Eastern Mediterranean, South East Asia, and Western Pacific [2]. America, South East Asia, and Western Pacific regions are the most seriously affected [1, 2]. In recent years, there is an increasing number of dengue infection cases detected predominantly in urban and semiurban areas and therefore has become a major international public health concern. Severe dengue has become a leading cause of hospitalization and death among children and adults in many regions, especially Asian and Latin American countries [3, 4].

In Vietnam, dengue was first recognized since the 1960s, thanks to the dengue epidemics in the Hanoi (North of Vietnam) and Cai Be (South of Vietnam). Recently, dengue has been reported to affect most provinces of the country [5], and the peak of infection is in June to October every year. Due to the wide geographic distribution of the mosquito vector and circulation of all four types of Dengue virus, dengue could rapidly spread across the country [6–9]. Therefore, the Viet Nam's National Dengue Control Program was established in 1999, and Vietnam has also been successfully recorded in controlling mortality from dengue fever [9].

Although the disease is now endemic in Vietnam, the knowledge of adult dengue virus infection is still limited and therefore requires a nationwide extensive analysis of clinical and epidemiological results. Such data will also provide useful information for establishing the dengue fever prevention program in Vietnam. In the early year of 2017, an outbreak of dengue fever transmitted throughout the country with much higher number of cases than in previous years. This study was undertaken to examine the clinical and laboratory profile of dengue infection in adult patients and to determine any new insights into the 2017 outbreak.

## 2. Materials and Methods

### 2.1. Population Study

The study included patients from 18 years old, diagnosed with dengue during outbreak of the disease from 1 January to 31 December 2017. Patients were recruited from the two largest centers for infectious diseases in Vietnam: National Hospital for Tropical Diseases (NHTD) in the North and Hospital for Tropical Disease (HTD), Ho Chi Minh City, in the South of Vietnam.

### 2.2. Study Design

This is a cross-sectional study. The case definition was based on compatible clinical history and examination based on WHO criteria, confirmed by positive for the NS1 antigen (rapid test, SD Bioline) or dengue IgM antibodies (rapid test, SD Bioline) or PCR method to detect dengue virus serotype. The PCR can detect dengue virus serotyping using specific primers and probe for each dengue serotypes from 1 to 4. All subjects were classified according to WHO guidelines 2009 [1]. We excluded the patients with confirm other acute infectious diseases such as measles, influenza, or chikungunya. Demographic data and details of clinical history and careful clinical examination were performed. Besides the routine test such as hematocrit, total leucocyte count, platelet count, liver enzyme (ALT and AST), blood urea, and serum creatinine, other investigations were performed according to the clinical conditions of the patients. Patients having nonspecific manifestations were grouped in expanded dengue syndrome category [2].

Sample size: we used a sample calculation formula for a descriptive study:

(1) $$\begin{array}{c} n=\frac{z_{1-\left(\alpha /2\right)}^{2}\;\left(1-p\right)}{p\varepsilon^{2}}, \end{array}$$

where *n* is the minimum sample size; *p* is the rate of severe dengue patients, estimated *p*=10%; *ε* is the relative error, we choose *ε*=18% (15–20%); and *z*_1_−*α*/2 is the reliability factor, with 95% confidence, *z*_1_−*α*/2 = 1.96.

The calculated sample size in this study was 1100 patients, at one study site. At each study site, based on a sample size of 1100 patients, the numbers of patients per month were randomly selected proportional to the number of hospitalized patients in the year. The total 2922 patients were selected for this study, including 1738 (59.5%) patients in NHTD site and 1184 (40.5%) patients in HTD site.

All statistical analyses were performed using the SPSS statistical version 17.0. Descriptive statistics like numbers and percentages were enumerated for all categorical variables. The chi-square (χ^2^) test was used to evaluate statistical differences in categorical variables between the groups. A *p* value <0.05 was considered significant.

The study protocol was approved by the ethical committee at NHTD, and all patients were provided informed consents.

## 3. Results

### 3.1. Demographic Characteristics

**Figure 1:**
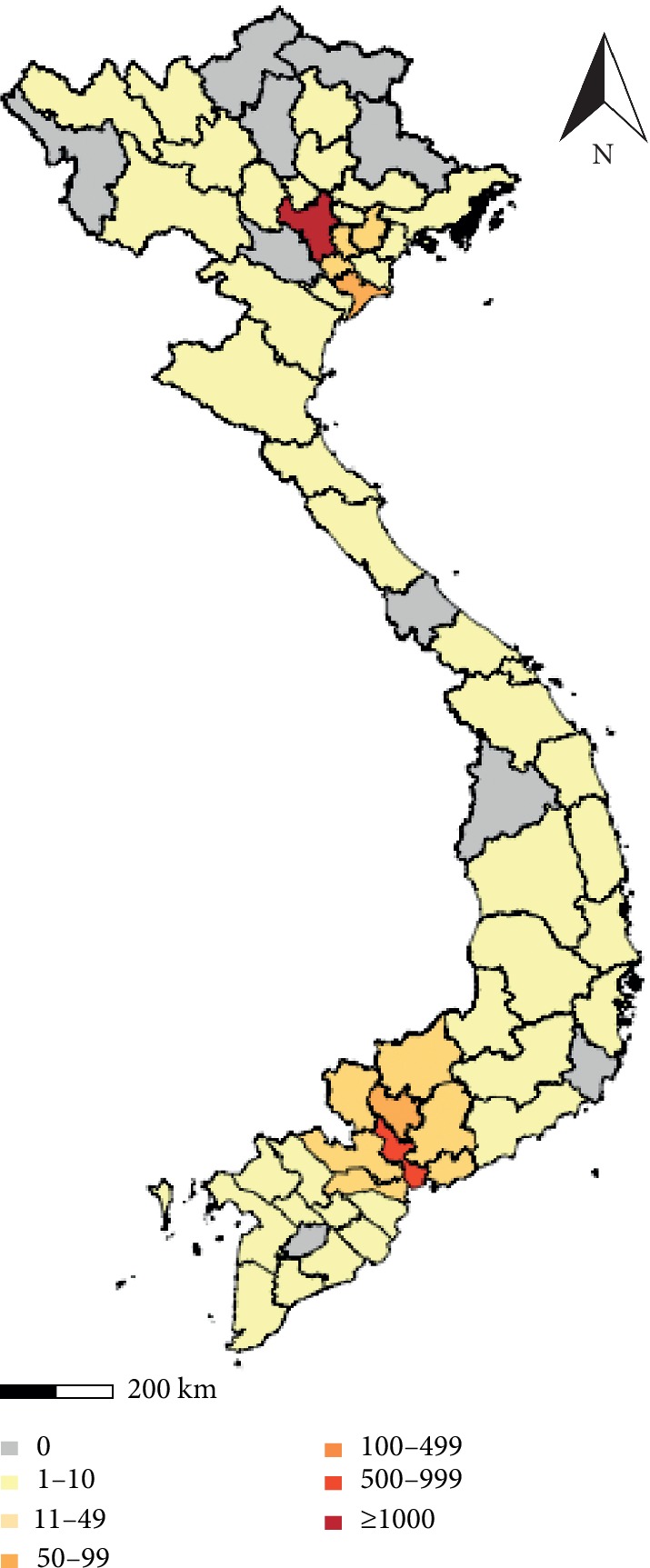
Map of dengue outbreak 2017 in Vietnam. Hanoi (in the North) and Ho Chi Minh city (in the South) are highlighted in red with higher number of dengue cases than other provinces.

Of the 2922 patients 1392 (47.6%) were male and 1530 (52.4%) were female. The rate male: female = 1 : 1.1. In the dengue outbreak in 2017, dengue patients were found in all ages, from 18 to over 80 years old, although most patients were less than 40 years old. Patients admitted hospital around the year, concentrated from August to December of that year, and inhabited in 53/63 (84.0%) provinces in Vietnam. Hanoi and Ho Chi Minh cities were the area with highest number of dengue patients. The map color in each province was in accordance with the number of dengue cases found (Figure 1). In this study, there were 184 (6.3%) pregnancy and 245/2922 (8.4%) patients with underlying diseases such as liver disease (chronic hepatitis and cirrhosis), kidney disease (chronic nephritis and renal failure), diabetes, hyperthyroidism, cardiovascular disease, and hypertension (Table 1).

### 3.2. Clinical Presentations

In this epidemic, the common manifestations were fever (96.9%), skin erythema, (69.7%), myalgia (48.7%), and hemorrhagic manifestation (48.4%). In addition, other signs of nonspecific infections were encountered with low frequency (Table 2).

### 3.3. Laboratory Findings in Patients with Dengue Fever

**Figure 2:**
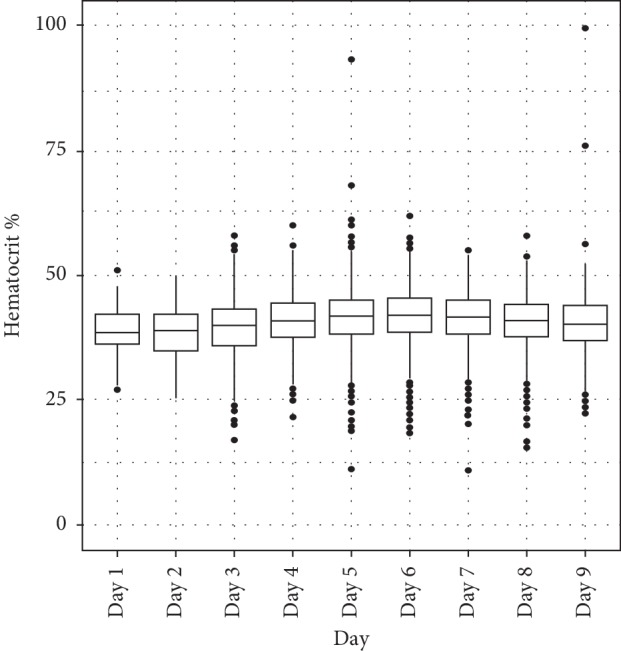
Variation of hematocrit according to days of illness in dengue patients.

**Figure 3:**
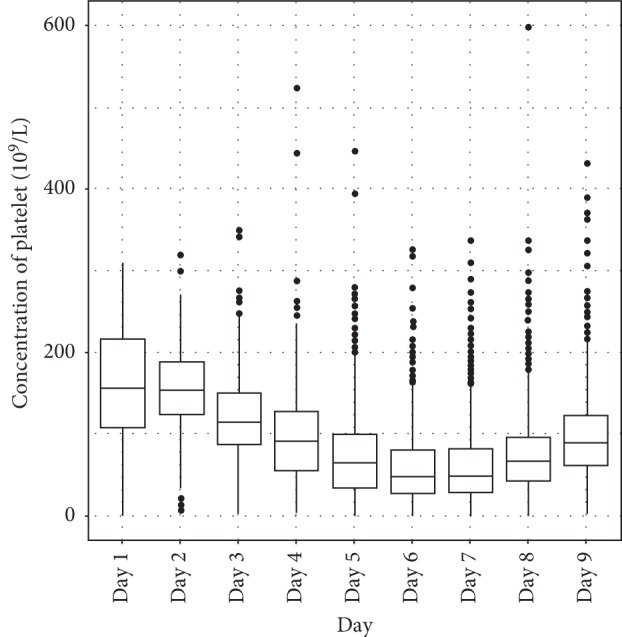
Thrombocytopenia according to days of illness in dengue patients.

From day 4 of fever, hematocrit began to rise and platelets began to drop below 100,000/mm3 and both tended to recover on the 9th day of the disease (Figures 2 and 3).

In this study, the proportion of patients with dengue fever: dengue with warning: severe dengue was 5 : 2.7 : 1, respectively. Among patients with severe dengue include severe plasma leakage and dengue shock (8.1%), severe organ impairment (2.5%), and severe bleeding (0.75%) (Table 3). The mortality rate in this study was 24/2922 (0.8%). Causes of death were shock, severe organ impairment, and severe bleeding. Outcome of dengue fever patients was related to a number of factors such as age and underlying diseases (Table 4).

## 4. Discussion

Vietnam is a developing country located in an area with tropical climate, where rainfall and temperature are favorable for mosquitoes to develop and spread dengue disease, especially in the South [2]. Large dengue outbreaks were reported elsewhere globally in 2016 [3]. Dengue fever has been recognized as a health problem in Vietnam [9]; however, the results of our study also showed some important issues of the 2017 dengue epidemic as follows.

### 4.1. Epidemiology and Demographics

#### 4.1.1. Geographically Dispersed

In Vietnam, the first cases of dengue fever were recorded in 1959 in the North and in the South in 1960. Until 1996, the disease was reported to spread to all provinces in Central and Southern Vietnam. However, the disease only occurred in 2/3 of the provinces and cities in the highlands and 15/23 (65%) of provinces and cities in the North which was not common in mountainous provinces [5]. This issue has been assessed by some studies as the spread of dengue fever is associated with climate characteristics in different areas in Vietnam [10, 11]. However, during the outbreak of dengue fever in 2017, dengue fever patients were present in 4/5 of the provinces, cities in the Highlands, and 19/25 (76%) of the provinces, cities in Northern. Dengue fever has occurred in the midlands and northern mountains, such as Son La and Lai Chau, where the economy and transport are growing rapidly. In this study, we identified the living address of patients based on medical records. It should be noted that not only the mosquitoes control but also human activities such as tourism and urbanization are the main factors contributing to the spread of dengue virus [1, 2, 12]. In the process of development of economic and traffic system [13], the movement of patients across regions is the most important factor that facilitates the widespread of dengue in Vietnam.

#### 4.1.2. Time of Patients Hospitalized in the Year and Age Distribution

Studies have documented dengue patients hospitalized year-round in the South and hospitalized only in the rainy season, from August to November, in the North [9, 11, 14], but in the outbreak 2017, patients with dengue in the North were also hospitalized around the year, including winter months (Table 1). It is possible that climate change with warming trend is a favorable factor for mosquitoes to develop and cause epidemics year-round not only in Southern Vietnam but also in the whole country [11, 13]. Most patients in this study admitted to hospital at around day 4 to day 6 of illness, which could be refer as “critical phase” or “with warning signs” according to Vietnam dengue prevention program guideline. Similarly, some studies have shown that dengue fever was only reported in patients under 60 years of age, focusing mainly aged under 40 due to expose to dengue virus in daily activities [6, 15]. Our findings showed that dengue fever has affected all ages, especially in the age group above 80 years, the oldest being 85-year-old. Along with the aging population, the issue of dengue infection was started to be seen more frequently in aging population in Vietnam.

### 4.2. Clinical and Laboratory

In this epidemic, the clinical manifestations and laboratory of the disease are similar to previous research results [8]. The most frequent symptom is fever, accounting for 97% patients in this study. The remaining 3% without fever is explained by the lack of fever evidence during disease progression; i.e., no temperature recording was found. However, comparing between the two areas, we found that, in the South, the clinical manifestations of dengue fever was more serious with the signs of hemorrhage, hepatomegaly, abdominal pain, and vomiting (Table 2), and the proportion of patients with warning signs was also higher (41% compared to 24.7%) (Table 3). This can be explained because in the South, patients appeared throughout the year and were infected by all 4 types of dengue viruses [6, 15]. However, the number of severely classified patients between the North and the South was similar (11.6% and 11.1%).

As with the earlier outbreak, hematocrit began to rise and platelets began to drop below 100,000/mm^3^ from day 4 of the disease and both tended to recover on day 9. The hematocrit according to illness day was highest in day 5 and day 6 of illness. Thrombocytopenia was found in dengue patients with lowest median platelet count on day 6 [1]. There were no difference between the North and the South of Vietnam in terms of hematocrit and platelet. The change of such two indexes is in accordance with dengue disease progression through 3 phases, as mentioned by many medical literature [1, 2].

The rate of shock was higher than in the South, while in the North, the rate of organ failure was higher (Table 3). There may be an association between organ failure with old age or underlying disease in dengue in adults.

#### 4.2.1. Outcome of Dengue Fever

There is a note in comparison with the previous reports of dengue fever, death mainly in the Southern and common in children [9], and this study shows that elderly people in Northern Vietnam should also be concerned. Results of this study showed that gender was not associated with the prognosis, but at higher ages, the risk of death was also higher (*p*=0.02). Moreover, the analysis of mortality rate of patients with and without underlying diseases shows that, in patients with underlying disease, the mortality risk is higher than the other group (*p*=0.0024).

Some studies have also reported that, in adults, the factors associated with severe dengue fever are age over 40 years [16–18], comorbidities [17, 18], and higher alanine aminotransferase (ALT) level [18, 19]. In this study, we could not rule out the relationship between mortality and aging population and underlying diseases. This issue needs a further study in the future. However, we could not differentiate the mortality rate among patients with early admission (before 4 days of illness) and late admission (after 4 days of illness).

#### 4.2.2. Limitations of the Study

First, the primary and secondary infection status of dengue patients was not included in this study. This may explain why there was a substantial difference in number of patients with warning signs in the South. Second, the classification between dengue fever, dengue fever with warning signs, and severe dengue fever sometimes has no clear boundaries. Third, in the context of an article, we have not been able to find out the relationship between the elderly, the underlying disease, and the risk of death. Finally, climate change could be one of the factors contributing to the epidemics; however, we could not discuss this important observation in more detail together with the mosquito index and the temperature of the North during the 2017 outbreak. This is a study conducted in the two biggest hospitals for tropical diseases in Vietnam, so it provides more information for dengue fever situation in Vietnam.

## 5. Conclusion

Dengue fever is becoming more serious medical issue in Vietnam. The disease affects all age groups and provinces nationwide. Without the dengue vaccination, it is suggested to continuously communicate and educate people about diseases prevention as well as vector control. In the future, more studies are requested to monitor dengue fever surveillance across the whole country and determine the biomarkers associated with prognosis, intervention, and treatment in order to reduce the mortality and morbidity.

## Figures and Tables

**Table 1:** Demographic of patients enrolled in the study.

	NHTD	HTD	Total
	*n* (%)	*n* (%)	*n* (%)
Total	1738(59.5)	1184(40.5)	2922 (100)

Gender			
Male	842 (48.4)	550 (46.6)	1392 (47.6)
Female	896 (51.6)	634 (53.4)	1530 (52.4)

Age			
≤20 years	184 (10.6)	139 (11.7)	323 (11.1)
21–30 years	652 (37.5)	570 (48.1)	1222 (41.8)
31–40 years	382 (22.0)	304 (25.7)	686 (23.5)
41–50 years	228 (13.1)	118 (10.0)	346 (11.8)
51–60 years	183 (10.5)	42 (3.5)	225 (7.7)
61–70 years	75 (4.3)	10 (0.8)	85 (2.9)
71–80 years	25 (1.4)	1 (0.1)	26 (0.9)
>80 years	9 (0.5)	0 (0.0)	9 (0.3)

Underlying diseases	166 (9.6)	79 (6.7)	245 (8.4)

Pregnant women	73 (4.2)	111 (9.4)	184 (6.3)

Months			
January	1 (0.05)	85 (7.2)	86 (2.9)
February	2 (0.11)	77 (6.5)	79 (2.7)
March	1 (0.05)	71 (6.0)	72 (2.5)
April	3 (0.17)	59 (5.0)	62 (2.1)
May	4 (0.2)	59 (5.0)	63 (2.2)
June	11 (0.6)	102 (8.6)	113 (3.9)
July	79 (4.5)	113 (9.5)	192 (6.6)
August	492 (28.3)	121 (10.2)	613 (20.9)
September	598 (34.4)	108 (9.1)	706 (24.1)
October	357 (20.5)	145 (12.2)	502 (17.2)
November	144 (8.3)	128 (10.8)	272 (9.3)
December	46 (2.6)	116 (9.8)	162 (5.5)

Day admission			
1–3 days of illness	443 (25.5)	328 (27.7)	771 (26.4)
Day 4–6 of illness	1136(65.4)	771 (65.1)	1907 (65.4)
>6 days	159 (9.1)	85 (7.2)	244 (8.4)

Distribute by regions			
North	19/25 (76.0%) provinces		53/63 (84.0%) provinces
Centre	12/14 (86.0%) provinces		
High land	4/5 (80.0%) provinces		
South	18/19 (95.0%) provinces		

**Table 2:** Clinical manifestations.

Clinical	NHTD	HTD	Total
	*n* (%)	*n* (%)	*n* (%)
Fever	1715 (98.7)	1116 (94.3)	2831 (96.9)
Skin erythema	1207 (69.4)	831 (70.2)	2038 (69.7)
Myalgia	980 (56.4)	442 (37.3)	1422 (48.7)
Bleed (any hemorrhagic manifestations)	688 (39.6)	725 (61.2)	1413 (48.4)
Generalized body ache/arthralgia	949 (54.6)	131 (11.1)	1080 (37.0)
Nausea and/or vomiting	317 (18.2)	552 (46.6)	869 (29.7)
Anorexia	196 (11.3)	365 (30.8)	561 (19.2)
Abdominal pain	126 (7.3)	369 (31.2)	495 (16.9)
Loose stools	117 (6.7)	255 (21.5)	372 (12.7)
Cough	72 (4.1)	221 (18.7)	293 (10.0)
Sore throat	33 (1.9)	93 (7.9)	126 (4.3)
Retroorbital pain	58 (3.3)	65 (5.5)	123 (4.2)
Nasal discharge	13 (0.7)	76 (6.4)	89 (3.0)
Hepatomegaly	8 (0.46)	53 (4.5)	61 (2.1)
Lymphadenopathy	3 (0.2)	46 (3.9)	49 (1.7)

Expanded dengue syndrome			
Bradycardia	6 (0.3)	43 (3.6)	49 (1.7)
Respiratory failure	11 (0.6)	41 (3.5)	52 (1.8)
Jaundice	4 (0.2)	44 (3.7)	48 (1.6)
Lethargic	11 (0.6)	43 (3.6)	54 (1.8)
Confuse	5 (0.3)	42 (3.5)	47 (1.6)
Convulsions	5 (0.3)	41 (3.5)	46 (1.6)
Other unusual neurological signs	12 (0.7)	40 (3.4)	52 (1.8)

**Table 3:** Clinical classification.

Clinical classification	NHTD	HTD	Total
	*n* (%)	*n* (%)	*n* (%)
Dengue fever	1108 (63.8)	567 (47.9)	1675 (57.3)
Warning signs of dengue	429 (24.7)	485 (41.0)	914 (31.3)
Severe dengue	201 (11.6)	132 (11.1)	333 (11.4)
Severe plasma leakage and dengue shock	129 (7.4)	109 (9.2)	238 (8.1)
Severe organ impairment	58 (3.3)	15 (1.27)	73 (2.5)
Severe bleeding	14 (0.8)	8 (0.68)	22 (0.75)

**Table 4:** Outcome in dengue cases.

	Mortality	*p*
Gender		
Male	10/1392 (0.72)	0.7
Female	14/1530 (0.92)	

Age		
≤40 years	10/2245 (0.44)	
41–60 years	8/563 (1.4)	0.02^*∗*^
≥60 years	6/114 (5.3)	

Severe dengue		
With underlying diseases	10/57 (17.5)	
Without underlying diseases	14/276 (5.1)	0.0024

^*∗*^ *p* value indicates the differences in terms of mortality rate among 3 age groups.

## Data Availability

The data used to support the findings of this study were supplied by National Hospital for Tropical Diseases (NHTD) in the North and Hospital for Tropical Disease (HTD), Ho Chi Minh City, in the South of Vietnam and so cannot be made freely available. Requests for access to these data should be made to the corresponding author. Data are available for researchers who meet the criteria for access to confidential data.

## References

[1] World Health Organization (2009). *Dengue: Guidelines for Diagnosis, Treatment, Prevention and Control (New Edition)*.

[2] World Health Organization, Regional Office for South-East Asia (2011). *Comprehensive Guidelines for Prevention and Control of Dengue and Dengue Haemorrhagic Fever (Revised and Expanded Edition)*.

[3] World Health Organization (2017). *Fact Sheet Dengue and Severe Dengue*.

[4] Cucunawangsih, Lugito N. P. H. (2017). Trends of dengue disease epidemiology. *Virology: Research and Treatment*.

[5] Ha D. Q., Huan T. Q. (1997). Dengue activity in Viet Nam and its control programme, 1997-1998. *Dengue Bulletin*.

[6] Ha D. Q., Ninh T. U. (2000). Virological surveillance of dengue haemorrhagic fever in Viet Nam, 1987–1999. *Dengue Bulletin*.

[7] Rabaa M. A., Simmons C. P., Fox A. (2013). Dengue virus in sub-tropical northern and Central Viet Nam: population immunity and climate shape patterns of viral invasion and maintenance. *PLoS Neglected Tropical Diseases*.

[8] Thai K. T. D., Phuong H. L., Thanh Nga T. T. (2010). Clinical, epidemiological and virological features of Dengue virus infections in Vietnamese patients presenting to primary care facilities with acute undifferentiated fever. *Journal of Infection*.

[9] WPRO—WHO Representative Office Viet Nam (2017). *Fact Sheet Dengue*.

[10] Vu H. H., Okumura J., Hashizume M., Tran D. N., Yamamoto T. (2014). Regional differences in the growing incidence of dengue fever in Vietnam explained by weather variability. *Tropical Medicine and Health*.

[11] Do T. T. T., Martens P., Luu N. H., Wright P., Choisy M. (2014). Climatic-driven seasonality of emerging dengue fever in Hanoi, Vietnam. *BMC Public Health*.

[12] Gubler D. J., Duane J., Gubler E. E. O. (2014). Dengue viruses: their evolution, history and emergence as a global PublicHealth problem. *Dengue and Dengue Hemorrhagic Fever*.

[13] Åström C., Rocklöv J., Hales S., Béguin A., Louis V., Sauerborn R. (2012). Potential distribution of dengue fever under scenarios of climate change and economic development. *Eco Health*.

[14] Thi Tuyet-Hanh T., Nhat Cam N., Thi Thanh Huong L. (2018). Climate variability and dengue hemorrhagic fever in Hanoi, Viet Nam, during 2008 to 2015. *Asia Pacific Journal of Public Health*.

[15] Quyen D. L., Le N. T., Anh C. T. V. (2018). Epidemiological, serological, and virological features of dengue in nha trang city, Vietnam. *The American Journal of Tropical Medicine and Hygiene*.

[16] Pinto R. C., de Castro D. B., de Albuquerque B. C. (2016). Mortality predictors in patients with severe dengue in the state of amazonas, Brazil. *PLoS One*.

[17] Temprasertrudee S., Thanachartwet V., Desakorn V., Keatkla J., Chantratita W., Kiertiburanakul S. (2018). A multicenter study of clinical presentations and predictive factors for severe manifestation of dengue in adults. *Japanese Journal of Infectious Diseases*.

[18] Mallhi T. H., Khan A. H., Sarriff A., Adnan A. S., Khan Y. H. (2017). Determinants of mortality and prolonged hospital stay among dengue patients attending tertiary care hospital: a cross-sectional retrospective analysis. *BMJ Open*.

[19] Trung D. T., Thao L. T. T., Vinh N. N. (2010). Liver involvement associated with dengue infection in adults in Vietnam. *The American Journal of Tropical Medicine and Hygiene*.

